# 

**Published:** 1815-10

**Authors:** 


### LECTURES.

The Lectures on Midwifery at the Middlesex Hospital, by Dr. Merriman, Physician-Accoucheur to that Hospital, and Consulting Physician-Accoucheur to the Westminster General Dispensary, will re-commence on Monday, October the 9th.

*School of Medicine in Ireland.*---The Winter Courses of Lectures on Anatomy, Physiology, Pathology, Surgery, Chemistry, Materia Medica, Institutes and Practice of Medicine, will commence on the 6th of November, at their respective hours.——Anatomical Demonstrations will commence the 1st of December.——Certificates of the professors in Trinity College, Dublin, are admitted in place of attendance in the Continental, English, and Scotch, Universities.

### BOOKS IN THE PRESS.

Rudiments of the Anatomy and Physiology of the Human Body, in a Series of Progressive Lessons, consisting of Several Tables, &c. accompanied with appropriate Explanations, designed for the use of Students of Medicine at the commencement of their Anatomical Enquiries. By T. J. Armiger, of the Royal College of Surgeons in London, &c. &c. late Demonstrator of Anatomy, at the London Hospital.

Messrs. Highley and Son will publish, in a few days, A Chemical Table, by Mr. Crowe, Surgeon, in the Royal Navy, exhibiting an Elementary View of Chemistry, intended for the use of Students and Young Practitioners in Physic, also to revive the Memory of more experienced Persons, being very convenient for hanging in Public and Private Libraries.

Mr. Carpue's Work on the Nasal Operation, with Plates, will appear in a few days.

Mr. J. B. Sharpe, Member of the College of Surgeons, is reprinting the Report of the Committee of the House of Commons on Madhouses; and, for the great convenience of the reader, has arranged each subject of evidence under its distinct head.

### MONTHLY CATALOGUE OF MEDICAL BOOKS.

Highley and Son's Catalogue of Medical Books, containing the most Modern and approved Works on Anatomy, Medicine, Surgery, Midwifery, Materia Medica, Chemistry, and Veterinary Surgery. To which are added, a complete List of the Lectures delivered in London, with their terms.

A Catalogue of an Extensive Collection of Books on Anatomy, Surgery, Medicine, Midwifery, Chemistry, &c. New and Second Hand; including a valuable Assortment of Medical Books recently imported from the Continent: sold by John Anderson, 40, West Smithfield, London. To which is added, a Complete List of the Lectures delivered in London, with their terms and hours of attendance, &c. Together with the Tables of the pay of the Medical Department of the Army, Navy, and East India Company's Service.

Reports of the Pestilential Disorder of Andalusia; by Sir James Fellowes, M. D. 8vo.

A Critical Inquiry into the Pathology of Scrofula, in which the origin of that Disease is accounted for on the new principles; and a new and much improved method is recommended and explained on the Treatment of it. By George Henning, M.D. 8vo.

Cases of Diseased Bladder and Testicle; by William Wadd, Surgeon; illustrated with Twenty-one Etchings by the Author. 4to. Callow.

Five Cases of Recovery from the Effects of Arsenic; by John Marshall. 8vo. Chapple.

A Treatise on Forensic Medicine, or Medical Jurisprudence; by O. W. Bartley, M. D. 12mo. Longman and Co.

An Outline of Mineralogy and Geology, intended for the use of those who may desire to become acquainted with the Elements of those Sciences. By William Phillips. 12mo. Phillips.

Sketches of the Medical School of Paris; including Remarks on the Hospital Practice, Lectures, Anatomical Schools, and Museums; and exhibiting the actual State of Medical Instruction in the French Metropolis. By John Cross. 8vo. Callow.

### *NOTICE TO CORRESPONDENTS.*

*We have been favoured with Communications from Drs. Buxton, Kinglake, Walker, and Messrs. Yeatman, S. J. E. &c. &c. &c.*

